# Management of vaginal mesh exposure: A systematic review

**DOI:** 10.1080/2090598X.2019.1589787

**Published:** 2019-04-04

**Authors:** Andrew Bergersen, Cameron Hinkel, Joel Funk, Christian O. Twiss

**Affiliations:** Department of Surgery, Division of Urology, University of Arizona College of Medicine, Tucson, AZ, USA

**Keywords:** Mesh exposure, mesh extrusion, pelvic organ prolapse, stress urinary incontinence, vaginal mesh

## Abstract

**Objectives**: To identify various predisposing factors, the clinical presentation, and the management of vaginal mesh-related complications, with special emphasis on mesh exposure and the indications for and results of vaginal mesh removal.

**Methods**: A systematic literature review was performed using a search strategy based on the Preferred Reporting Items for Systematic Reviews and Meta-analyses criteria. PubMed was queried for studies regarding aetiology, risk factors, and management of vaginal mesh exposure from 1 January 2008 to June 2018. Full-text articles were obtained for eligible abstracts. Relevant articles were included, and the cited references were used to identify relevant articles not previously included.

**Results**: A total of 102 abstracts were identified from the PubMed search criteria. An additional 45 studies were identified based on review of the cited references. After applying eligibility criteria and excluding impertinent articles, 58 studies were included in the final analysis.

**Conclusion**: Numerous studies have found at least some degree of symptomatic improvement regardless of the amount of mesh removed. Focal areas of exposure or pain can be successfully managed with partial mesh removal with low rates of complications. With partial mesh removal, many patients will ultimately require subsequent mesh removal procedures. For this reason, complete mesh excision is an alternative for patients with diffuse vaginal pain, large mesh exposure, and extrusion of mesh into adjacent viscera. However, when considering complete mesh removal, it is important to counsel patients regarding possible complications of removal and the increased risk of recurrent stress urinary incontinence and pelvic organ prolapse postoperatively.

**Abbreviations**: MUS: midurethral sling; OR: odds ratio; POP: pelvic organ prolapse; PRISMA: Preferred Reporting Items for Systematic Reviews and Meta-analyses; SUI: stress urinary incontinence; TOT: transobturator; TVT: tension-free vaginal tape

## Introduction

The use of synthetic material for surgical correction of pelvic organ prolapse (POP) and stress urinary incontinence (SUI) has become increasingly common. Synthetic midurethral sling (MUS) placement is now widely regarded as the standard of care for SUI [1]. The use of the MUS has been shown to have significantly higher rates of subjective improvement, as well as subjective and objective cure rates, compared to conservative management of SUI [2]. In a recent large review, 80% of women with SUI were cured or experienced significant improvement in their symptoms after placement of a retropubic or transobturator (TOT) MUS [3].

Despite its effectiveness, the use of mesh has been associated with several complications, including obstructive voiding symptoms, vaginal or pelvic pain, dyspareunia, recurrent UTI, recurrent SUI, and mesh exposure [4,5]. Mesh used for POP repair is subject to similar complications [6]. The MUS complication rate was reported to be as high as 25% at 5-years follow-up in a recent study [4]. Synthetic sling failure is seen in 12–20% of patients [7]. The rate of postoperative pelvic pain after placement of transvaginal tape or mesh varies from 0% to 30% [8,9]. Whilst mesh complications may be asymptomatic, pain has been shown to be amongst the most common complications in those seeking treatment.

In the present review article, we identify various predisposing factors, the clinical presentation, and the management of mesh-related complications, with special emphasis on mesh exposure and the indications for and results of mesh removal.

## Mesh terminology

Based on consensus statements from the ICS and the International Urogynecological Association (IUGA), the term ‘exposure’ will be used to describe vaginal mesh visualised through separated vaginal epithelium and ‘extrusion’ will refer to the passage of mesh out of a body structure or tissue, such as the presence of mesh within the bladder or urethra [10]. It is important to be cognisant that these terms do not imply the actual cause of the improper location of the mesh graft.

## Methods

A systematic review was performed using PubMed to identify relevant studies. The search was restricted to publications in English. Literature review was conducted in June 2018. The review of the studies was conducted independently by two authors (A.B. and C.H.).

To capture the entirety of the topic of vaginal mesh exposure and subsequent management, two separate searches were conducted. The first literature search used a free text protocol with the following search terms: ‘urinary incontinence mesh’, ‘SUI mesh’, ‘SUI surgery’, ‘suburethral slings adverse effects’, ‘mesh exposure/extrusion/erosion’, ‘postoperative mesh-related complications’, ‘mesh exposure/extrusion/erosion treatment’. The second literature search used the following search terms: ‘POP mesh’, ‘POP mesh exposure/extrusion/erosion’, ‘mesh-related pelvic pain’, ‘POP mesh postoperative complications’, ‘POP mesh exposure/extrusion/erosion treatment’. Literature review was further refined based on the following restrictions: female gender, middle aged and elderly patients, and studies from the time period 2008–2018.

**Figure 1.**
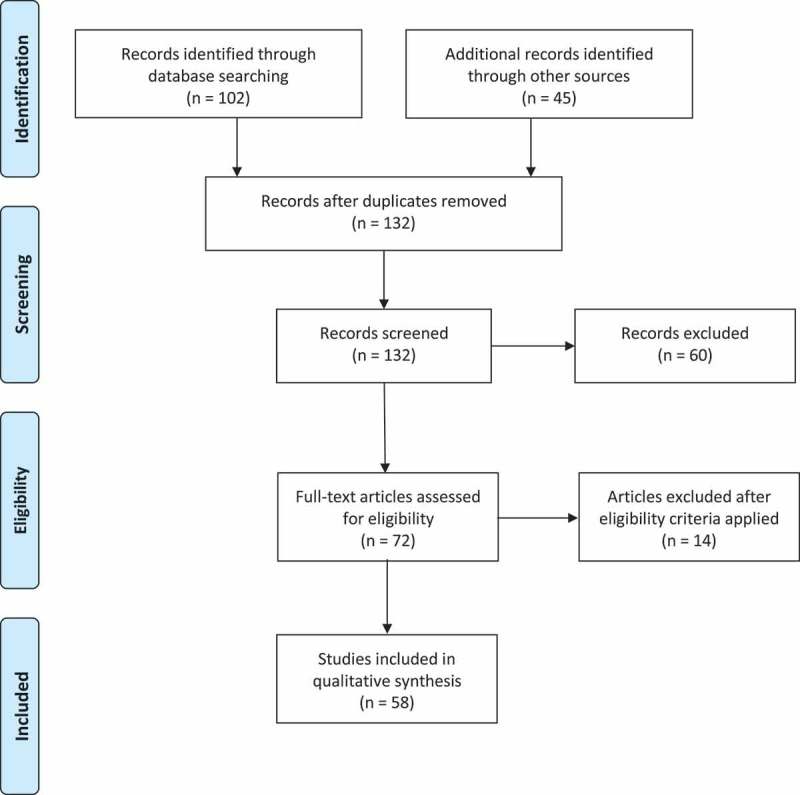
PRISMA flow diagram of study selection.

Article selection was based on the Preferred Reporting Items for Systematic Reviews and Meta-analyses (PRISMA) criteria strategy. The study selection process can be seen in the flow diagram in Figure 1.

Given the paucity of high-quality studies on the topic of management of mesh exposure, a broad range of manuscript types were considered for inclusion in the review. Review articles, commentaries, case reports, and small case series were included if they were determined to have information relevant to the topic. The eligibility criteria are listed in Table 1.

**Table 1.** Eligibility criteria for paper selection.

Inclusion criteria	Exclusion criteria
Study pertaining to the aetiology of mesh exposure, risk factors for mesh exposure, presentation of mesh exposure, and management of mesh exposure	Studies not pertaining to risk factors, management, or aetiology of mesh exposure
English language	Full-text unable to be obtained
Published from 1 January 2008 to June 2018	
Full-text published article	

Once abstracts were selected, the full-text articles were obtained. Review of the full-text articles included review of the cited references and significant papers that were not previously included were added.

## Results

A total of 102 abstracts were identified from the PubMed search criteria. An additional 45 studies were identified based on review of the cited references. After applying eligibility criteria and excluding impertinent articles, 58 studies were included in the final analysis.

### Clinical presentation of mesh exposure

The clinical presentation of mesh exposure can be quite variable. Signs and symptoms of mesh exposure include vaginal or pelvic pain, vaginal discharge or bleeding, odour, recurrent infection, abscess development, dyspareunia, or pain experienced by the sexual partner [11]. Pain is the most common presenting symptom. However, it is also possible that patients may present with asymptomatic mesh exposure.

### Risk factors for mesh exposure

There are many risk factors that predispose certain patients to mesh exposure. A summary of the risk factors for mesh exposure are listed in Table 2. Cigarette smoking has been found to be significantly associated with a higher rate of mesh exposure [12]. The technique used in placement of prolapse mesh has been shown to increase the risk of mesh exposure in certain cases. For example, using a combined vaginal and abdominal approach during a sacrocolpopexy increased the rate of mesh exposure from 4% (10/243) in the abdominal-only group to 20% (six of 30) in the combined group [13]. Increasing age has been reported as a risk for mesh exposure as well [11]. Other risk factors include diabetes, surgeon experience, and proper training in pelvic organ reconstructive procedures [14–16].

**Table 2.** Risk factors for vaginal mesh exposure.

Risk factor for mesh exposure	References
Patient-related factors	
Smoking	[11,12,15]
Diabetes mellitus	[11]
Patient age	[11]
Surgery-related factors	
Postoperative urethral dilatation	[14]
Excessive sling tensioning	[14]
Surgeon experience	[11,15]
Combined vaginal and abdominal approach for mesh placement	[13]
Inverted ‘T’ colpotomy	[11,17]
Concomitant hysterectomy	[11,17]

Concomitant procedures performed during the time of mesh placement have also been found to contribute to the rate of mesh exposure. Collinet et al. [17] reported in their series of 277 patients that concomitant hysterectomy (odds ratio [OR] 5.17) and inverted ‘T’ colpotomy (OR 6.06) were found to significantly increase the risk of mesh exposure. Ganj et al. [18] found that concomitant transvaginal hysterectomy and intraoperative bladder injury increased the risk of subsequent mesh exposure. The more extensive tissue dissection and manipulation associated with both concomitant hysterectomy and bladder injury are thought to contribute to the increased risk of mesh exposure in such cases [18]. Additionally, concomitant sling and POP mesh placement has been found to increase the risk of subsequent mesh exposure [19].

The type of mesh, whether MUS or POP mesh, also affects the risk of developing mesh exposure. Mesh-related complications have been found to be more common with use of POP mesh compared to that used for SUI repair [20].

### Aetiology of mesh exposure

Mesh exposure rates range from 2% to 30% following placement for repair of POP or for surgical correction of SUI [11,21–24]. The exact aetiology for mesh exposure remains unclear and there are probably several different factors that play a role. Errors during placement are thought to be an important cause. Margulies et al. [25] identified mesh folding at the time of mesh excision surgery in 69% of patients in their series presenting for mesh removal primarily due to vaginal pain. In a series of 90 patients presenting for mesh removal, Crosby et al. [26] reported that mesh was not found to be lying flat or tension free in 70% of patients. It remains difficult to determine if the folding observed at the time of mesh removal is due to errors during placement or if it is related to mesh contraction during the healing process, but it appears to be associated with mesh pain and exposure.

Another theory proposed to account for the aetiology of mesh exposure is the idea of mesh contraction. Vaginal mesh contraction was first described by Feiner et al. [27] in 2010 in their series of 17 patients who presented with significant vaginal pain and were found on examination to have a palpable, tender area of mesh contraction. Mesh contraction results in an area of bunched mesh under increased tension. They found this to be associated with a higher than expected rate of mesh exposure (53%). The vaginal pain and increased risk of mesh exposure is probably related to the excessive tension placed on the fixed mesh arms relative to the main body of the graft, resulting in bunching of the body of the graft. In a study of 684 patients undergoing transvaginal mesh placement for POP, Caquant et al. [28] found that a total of 80 patients (11.7%) had mesh retraction and 52.6% of those were associated with concomitant mesh exposure.

The type of mesh material used in the repair has been investigated as a possible factor influencing the rate of mesh exposure. It is already well established that small mesh pore sizes lead to increased mesh-related complications and that type 1 mesh is the recommended type for vaginal reconstructive surgery [29]. However, type 1 polypropylene mesh is offered in different weaves and fibre sizes (‘mesh weight’) [29]. Notwithstanding, Moore et al. [21] reported no statistically significant difference in exposure rates between type I polypropylene mesh and a lightweight version of the type I polypropylene mesh.

The route of sling placement, whether TOT or retropubic, remains indeterminate with regards to increased risk of mesh exposure. Kokanali et al. [16] reported that of 61 patients (4.2%) with sling erosion, 41 (67.2%) had prior TOT placement compared to 20 (32.8%) after tension-free vaginal tape (TVT). The overall rate of sling exposure in this study was 4.7% in the TOT group and 3.5% in the TVT group, a difference that was found to be statistically significant. However, Linder et al. [30] found retropubic sling placement to be predictive of exposure on multivariate analysis in a cohort of 2123 patients with prior sling placement.

### Conservative management

Conservative management consists of observation alone, use of topical oestrogens or antiseptics, systemic or topical antibiotics, and office-based trimming of the exposed material [31]. Conservative therapy is often recommended as a first-line option for focal areas of mesh pain or small areas of mesh exposure. The choice of management route is based on several factors, including the size and location of the exposure, the involvement of adjacent organs or structures, and the patient’s symptoms. Additionally, the index procedure and resultant complications factor into the decision to pursue conservative or operative management as well.

Conservative management may be attempted for asymptomatic mesh exposure. Initial management of minimally symptomatic or asymptomatic, focal areas of mesh exposure often consists of topical oestrogen therapy with or without antibiotics. The size of the mesh exposure factors heavily into the decision to pursue conservative management. It has been found that small exposures (<0.5 cm) can be managed conservatively [20]. Larger exposures (up to 4 cm) are unlikely to heal with conservative management and will typically require surgical management [32].

### Outcomes for conservative management

In a series involving both prolapse and sling mesh, failure of conservative methods for managing mesh exposure (consisting of oestrogen cream, antibiotics, and/or physical therapy) was reported in 63% [33]. Other groups have reported similarly high failure rates for conservative management [31]. Ultimately, many women who undergo conservative management will require surgical intervention. Abbott et al. [34] found that of 347 patients presenting with POP mesh or sling complications, 51% were initially managed conservatively. Of those treated conservatively, 59.3% went on to require surgical intervention. Abdel-Fattah et al. [24] reported on a series of 289 patients presenting with mesh-related complications and found a 10% exposure rate; 97% of these patients were initially managed conservatively, but all ultimately required surgical intervention.

The index procedure factors into the ultimate success of conservative management. Patients who underwent transvaginal placement of POP mesh are more likely to experience pain and dyspareunia compared to patients who underwent sacrocolpopexy, complications of which are more likely to include vaginal discharge and bleeding [33]. Additionally, patients presenting with mesh exposure are more likely to report vaginal bleeding and discharge rather than vaginal or pelvic pain [33].

Office-based mesh trimming remains challenging due to patient discomfort and difficulty visualising the area of mesh exposure. Most women will require formal exploration and excision in the operating room [34].

### Endoscopic management

Endoscopic management may be an option for patients with urethral or bladder perforation/extrusion of mesh material. The existing data for the endoscopic approach is limited to small case series. Techniques for endoscopic management of intravesical mesh include cystoscopic resection with endoscopic scissors, holmium laser, and transurethral resection using diathermy. One method utilises forceps passed alongside the cystoscope to grasp the mesh fibres and then using endoscopic scissors to cut the fibres close to the mucosa [14]. An alternative method described using a holmium laser applied to the mesh as close to the mucosa as possible [35].

#### Outcomes for endoscopic management

Success rates for endoscopic management of urethral extrusions have been found to be ~50% [14,36]. Other studies have shown similar success rates [37]. Doumouchtsis et al. [35] reported in their series utilising the holmium laser for repair of mesh extrusion that only one in four patients was symptom free and without endoscopic recurrence at the end of 2 years of follow-up. With regards to extrusion of mesh into the bladder, outcomes closely mirror those seen for urethral extrusion. Patients often require subsequent procedures, either repeat attempts at endoscopic excision or formal open excision of the area of extrusion [35,37].

### Partial mesh removal

#### Indications for partial mesh removal

Partial mesh excision is typically the initial procedure performed in patients presenting with limited areas of mesh exposure and mild symptoms. Focal areas of mesh exposure can be excised whilst leaving the remainder of the mesh implant intact. The mesh material itself has not been found to cause a carcinogenic or immune response after implantation, supporting the idea that asymptomatic mesh can be left in place [38].

Another common indication for partial mesh removal is the concern for surgical complexity in removing the arms of the graft. In a retrospective review of 374 patients undergoing complete mesh removal from one or multiple vaginal compartments, Pickett et al. [39] reported higher rates of intraoperative blood loss, as well as an increased transfusion rate in patients undergoing multi-compartment mesh removal.

#### Outcomes of partial mesh removal

Partial excision has been reported to have complete resolution rates as high as 71–95% for patients presenting with mesh exposure or urinary retention and no other symptoms [26,40]. For patients presenting with pain and/or dyspareunia, complete resolution is more difficult to achieve, and the rates of resolution vary widely in the literature, from 46% to 89% [26,41–44]. The rates of symptom resolution for pain vs mesh exposure are detailed in Table 3. In a small cohort of 21 patients presenting with complications following transvaginal mesh kit placement, Jeffery et al. [45] reported complete resolution of pain and dyspareunia in 20 (95%) patients following partial mesh removal at 24-months follow-up. However, Crosby et al. [26] found pain to be the most difficult symptom to manage, reporting only a 50% complete improvement of pain for patients presenting with pain.

**Table 3.** Symptom resolution rates for pain versus mesh exposure.

Reference	*N*	Symptoms prompting mesh removal, *n* (%)	Follow-up, months	Complete resolution rate, %	Pain resolution vs exposure resolution, %
Crosby et al. [26]	84	Mesh exposure 56 (62), Pain 58 (64), Dyspareunia 43 (48)	4 (median)	51 complete resolution	Pain: 49 Exposure: 95
Skala et al. [41]	54	Vaginal and/or pelvic pain 36 (66.7), Mesh exposure 30 (55.6), Vaginal discharge 26 (48.1)	3 (mean)	52 complete resolution (6 lost to follow-up)	Pain: 63.9 Exposure: 83.3
Marcus-Braun et al. [42]	10	Pelvic pain 10 (100)	6 (mean)	50 complete resolution	Pain: 50 Exposure: NA
Ridgeway et al. [43]	19	Pain 6 (31.6), Dyspareunia 6 (31.6), Recurrent POP 8 (42.1), Mesh exposure 12 (63.2), Vesico-vaginal fistula 3 (15.8)	33 weeks (median)	78.9 complete resolution	Pain: 83 Exposure: 100
Hurtado et al. [44]	12	Pelvic and/or vaginal pain 12 (100), Vaginal bleeding/discharge 4 (33.3), Dyspareunia 5 (41.7), Mesh exposure 9 (75)	3.4 (mean)	41.7 complete resolution	Pain: 50 Exposure: 100
Jeffery et al. [45]	21	Pain and/or dyspareunia 18 (85), Exposure 5 (24), POP 9 (43)	12 (mean)	76.2 complete resolution	Pain: 88.9 Exposure: 100

There is little data about the rate of recurrent symptoms, either POP or SUI, after partial mesh removal. In a series of 117 patients who underwent MUS removal, 33% of patients developed significant SUI requiring a subsequent anti-incontinence procedure. No significant differences were found between groups based on the type of sling (retropubic vs TOT) or amount of mesh removed (complete vs partial) [46].

Many patients who undergo an initial attempt at partial mesh removal often require subsequent procedures for further mesh removal. This can be due to the lack of resolution of presenting symptoms or recurrent mesh exposure. In a study by Tijdink et al. [33], a third of patients presenting to their centre for mesh-related complications had had prior attempts at mesh removal and ultimately required further mesh excision. In a series of 111 patients referred for mesh-related complications, Hansen et al. [47] performed mesh excision in 85 patients, 34 (40%) of whom had undergone previous mesh excision. Feiner et al. [27] reported 18% of patients in their series required subsequent complete mesh removal due to persistent vaginal pain after partial removal. Abbott et al. [34] reported that 21% of patients in their series required two or more mesh excision procedures and 8% of patients required three or more surgeries. In a cohort of eight patients presenting with persistent thigh pain after TOT sling placement, thigh dissection and graft removal was performed with all patients experiencing improvement in pain symptoms [48]. Numerous studies have shown that multiple mesh removal procedures are often necessary to achieve symptom alleviation [25,43].

Patients undergoing subsequent removal of additional mesh have reported further symptomatic improvement [34,47]. However, repeat mesh removal surgery is typically more challenging than the initial attempt. Patients are at increased risk of visceral injury and haemorrhage due to the presence of densely adherent mesh and the loss of surgical planes [27]. Additionally, re-approximation of the vaginal epithelium may be more difficult due to the increased fragility of the tissue and the presence of scarring.

### Complete mesh removal

#### Indications for complete mesh removal

Complete mesh removal consists of the intention to perform total mesh removal, or as much as possible when considering mesh arms passing through difficult areas to expose, including the obturator space, ischioanal fossa, or sacrum [33].

Complete mesh excision is more likely to be considered in the setting of vaginal/pelvic pain, large areas of exposure, severe symptoms, and/or involvement of the bladder or bowel [33,45,49]. Additionally, complete excision is performed more frequently in the case of POP mesh compared to patients whose index surgery was sling placement alone [34].

Another possible indication for complete mesh excision is infection of the graft material. Risk of infection is an inherent risk with any implant procedure. Colonisation of mesh material appears to be very common, but the role this plays in development of mesh-related complications remains unclear. Numerous studies have reported very low infection rates following uncomplicated vaginal mesh implantation [50,51]. In a series of 107 patients, Mellano et al. [52] found no difference in culture results from women presenting with delayed-onset pain vs acute pain, vaginal mesh exposures vs no exposures, or recurrent UTIs. This finding makes it difficult to predict with certainty that recurrent UTIs will resolve after complete mesh removal.

### Outcomes of complete vs partial removal, including complications

Postoperative complications include haematoma, UTIs, urinary retention, wound infection, subcutaneous abscess, fistula formation, and ureteric obstruction [33]. Reported intraoperative complications during complete mesh removal procedures include bowel injury, ureteric injury, bladder injury, and haemorrhage [33]. Given that complete mesh excision is more often performed for complicated cases of mesh-related complications, including mesh extrusion into nearby organs and fistula formation, complications of these surgeries can be severe. Postoperative complications include recurrent POP or SUI, wound infection, persistent vaginal or pelvic pain, and fistula formation. In a series of 277 patients, Rac et al. [53] reported a total of 155 complications in 131 patients after mesh excision surgery, only 14 (9.0%) of which occurred perioperatively. The most frequent complications were UTIs and vaginal yeast infections in 37 patients (23.9%), followed by *de novo* SUI in 31 patients (20.0%), *de novo* urge UI in 11 patients (7.1%), and *de novo* POP in seven patients (4.5%). The perioperative complications included seroma development in one patient (0.6%), bowel injury in three (2%), ureteric injury in two (1%), respiratory failure in one (0.6%), and iliac vein injury in one (0.6%). This is consistent with other reports of peri- and postoperative complications after mesh excision surgery, although the data are limited [39,54]. Overall, most complications tend to be minor and can typically be managed conservatively.

Some degree of symptom resolution is common following any amount of mesh excision. Tijdink et al. [33] found no difference in symptom resolution when comparing complete to partial mesh excision in patients who underwent either partial or complete removal for mesh-related complications (including mesh exposure, vaginal bleeding/discharge, dyspareunia, vaginal pain, recurrent UTIs, defaecation problems, and dysfunctional voiding), although five out of the six patients not experiencing any symptom improvement in this study underwent partial excision. Similar results were found in the series by Crosby et al. [26].

Regardless of the amount of mesh removed, pain remains a difficult symptom to alleviate. The degree to which symptom improvement relies on the amount of mesh removed remains unclear. In a series of 306 patients who underwent urethral sling and/or transvaginal mesh removal, Rogo-Gupta et al. [49] reported an overall improvement rate of 80% after removal; however, only 41% of patients reported ≥90% improvement. Hokenstad et al. [55] found complete mesh excision to be an independent predictor of successful patient-reported outcomes after excision of transvaginally placed mesh.

There are limited data about the impact of the amount of mesh removed on recurrent SUI after MUS removal or recurrent POP after removal of transvaginal POP mesh. Ramart et al. [46] found that complete mesh removal did not increase the rate of recurrent SUI after MUS removal compared to partial removal. However, Jambusaria et al. [56] found in their cohort of 245 patients with prior MUS that in patients presenting for mesh removal due to mesh exposure, complete sling excision resulted in significantly greater rates of postoperative SUI and repeat surgery for recurrent SUI. Although, in the same study, in patients presenting for excision due to pain along the sling, there was no statistically significant difference in rates of postoperative SUI or symptom improvement between the two groups [56]. A factor that may account for the difference in recurrent UI or POP after mesh removal is the definition of complete vs partial mesh excision. Misrai et al. [57] reported no difference in rates of recurrent SUI following complete or partial sling excision, but both partial and complete excision in their report included removing the entire vaginal portion of the sling.

The rate of recurrent POP after mesh removal varies throughout the literature. Tijdink et al. [33] reported a 12% recurrence rate and that POP recurrence was found to be more common in patients undergoing complete mesh removal compared to partial excision. Rawlings et al. [58] reported a 15% rate of recurrent POP after mesh removal. Marcus-Braun and von Theobald [40] reported a recurrent POP rate of 19%. Further, the rate of subsequent POP repair has been shown to range from 0% to 17% [58]. Again, the wide variability in rates of recurrent POP may be related to the method of defining recurrent POP and how much of the original mesh graft was actually removed. Definitions vary based on degree of bother, region of POP, and grade of POP, with a common, but not universal, threshold of Pelvic Organ Prolapse Quantifications System (POP-Q) Stage ≥II [58].

## Algorithm

**Figure 2.**
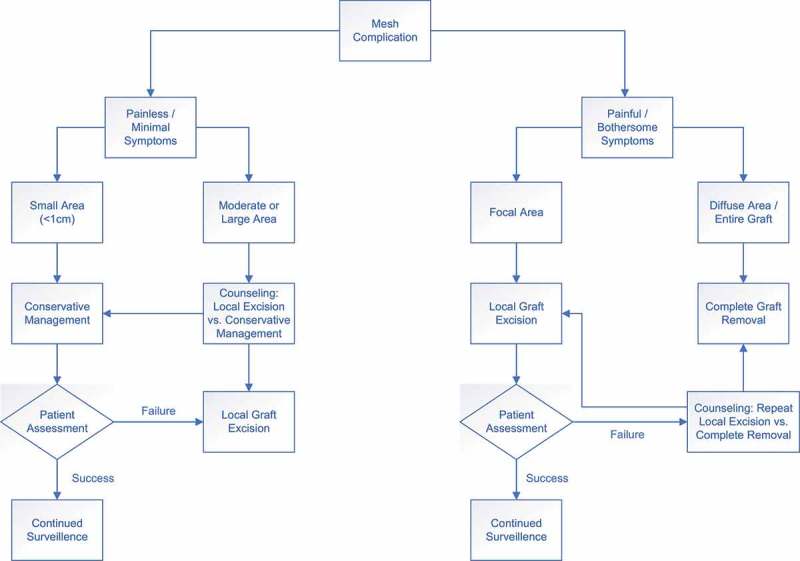
Algorithm for the management of mesh exposure.

Based on the findings of the present literature review, we propose an algorithm for the management of mesh pain and exposure, which can be seen in Figure 2. Patients are distinguished first based on whether the mesh complication is painful/symptomatic or asymptomatic. In patients with minimal symptoms, small mesh exposures are offered conservative management (oestrogen/observation), whereas larger exposures are candidates for elective local excision after counselling regarding low success rates of conservative management and risks of mesh removal. Patients with pain/bothersome symptoms are candidates for surgical mesh excision. Patients presenting with a focal problem with the graft (pain with or without concurrent mesh exposure) are offered partial mesh excision and re-assessed. Failures of local excision are counselled regarding the option of proceeding to complete removal of the remaining mesh vs continued attempts at local excision and the inherent risks of both approaches. Patients presenting with diffuse pain over the entire graft are offered the option of complete excision to provide the greatest probability of symptom improvement and avoidance of multiple failed interventions. With this approach, the risks of recurrent SUI or POP should be discussed in addition to risks of the procedure.

## Conclusion

Vaginal mesh use is associated with a number of potential complications, the most common of which are mesh exposure/extrusion, vaginal pain, and dyspareunia. Conservative measures are often attempted initially for management of small, minimally symptomatic areas of mesh exposure. However, for symptomatic patients, surgical intervention, which can include partial or complete mesh excision, is often necessary. Numerous studies have found that there is at least some degree of symptomatic improvement regardless of the amount of mesh removed. Focal areas of exposure or pain can be successfully managed with partial mesh removal with low rates of complications. With partial mesh removal, a subset of patients will ultimately require subsequent mesh removal procedures. For this reason, complete mesh excision may be an alternative option for patients with diffuse vaginal pain, large mesh exposure, and extrusion of mesh into adjacent viscera. However, when considering complete mesh removal, it is important to counsel patients regarding possible complications of removal and the increased risk of recurrent SUI and POP postoperatively. It is especially important to counsel patients that mesh removal may not completely alleviate symptoms as mesh-related pelvic pain is likely multifactorial in nature and can be very difficult to treat.
